# Coronation Honours

**Published:** 1911-06-24

**Authors:** 


### CORONATION HONOURS.

The list of Coronation Honours was issued on Monday night, and contains the names of many distinguished men in the medical world. The following is the complete list:—

IRISH PRIVY COUNCILLOR.

Michael F. Cox, Esq., M.D.

BARONETS.

Sir Charles Bent Ball, M.D., F.R.C.S.I. Regius Professor of Surgery of Dublin University, and hon. surgeon to the King in Ireland.

Henry Trentham Butlin, Esq. President of the Royal College of Surgeons, consulting surgeon to St. Bartholomew's Hospital.

James Frederic Goodhart, Esq., M.D. Consulting physician to Guy's Hospital.

William Osler, Esq., M.D., F.R.S. Regius Professor of Medicine in the University of Oxford.

Sir William Thornley Stoker, M.D. Dublin physician and surgeon, Inspector for Ireland under the Vivisection Act.

KNIGHTS.

Richard Barter, Esq. Leading hospital worker in Cork.

Anthony Alfred Bowlby, Esq., C.M.G., F.R.C.S. Surgeon to St. Bartholomew's Hospital. Surgeon to King Edward VII.'s Household from 1904-10.

Dr. Richard Brayn. Late Medical Superintendent, Broadmoor Asylum, a Home Office expert in lunacy.

Alexander Dempsey, Esq., M.D. Is ex-President of the Ulster Medical Society, and a leading Belfast practitioner.

Frederic William Hewitt, Esq., M.V.O., M.D., M.R.C.S. Anæsthetist to the King.

W. S. McCormick, Esq., LL.D. Done valuable work for the medical schools of Scotland.

Joseph McGrath, Esq., Secretary of the National University, Dublin.

Ernest Schiff, Esq. The founder of the Home for Surgical Convalescents at Swanley.

Frederick C. Wallis, Esq., M.B., F.R.C.S. Surgeon to Charing Cross, Grosvenor, and St. Mark's Hospitals.

The following additional list contains the Honours for the Services, the Colonies, and India:—

TO BE HONORARY SURGEON-GENERAL.

The Honourable Sir Frederick William Borden, K.C.M.G., M.D., M.P., Honorary Colonel Canadian Army Medical Corps, Minister of Militia and Defence Dominion of Canada.

KNIGHT COMMANDERS OF THE BATH.

Major Ronald Ross, C.B., F.R.C.S. Professor of Tropical Medicine at Liverpool University and the Liverpool School of Tropical Medicine. Entered Indian Medical Service in 1881, and in 1895 undertook experimental verification of mosquito theory of malaria. In 1902 was awarded the Nobel Prize for Medicine.

Benjamin Arthur Whitelegge, Esq., C.B., M.D., Chief Inspector of Factories.

Inspector-General of Hospitals and Fleets Doyle Money Shaw, C.B.

Inspector-General of Hospitals and Fleets Thomas Desmond Gimlette, C.B.

Surgeon-General Adam Scott Reid, C.B., Indian Medical Service (Retired).

Surgeon-General William Launcelotte Gubbins, C.B., M.V.O., K.H.S., Director-General Army Medical Service.

Inspector-General of Hospitals and Fleets Charles Cane Godding.

Inspector-General of Hospitals and Fleets Arthur William May.

Inspector-General of Hospitals and Fleets Howard Todd.

Fleet Surgeon Percy William Bassett-Smith.

Surgeon-General James Gaussen MacNeece, Principal Medical Officer, 8th (Lucknow) Division, India.

Surgeon-General George Winsor Robinson, Principal Medical Officer, Aldershot Command.

Colonel Charles Fancourt Willis, Indian Medical Service, Principal Medical Officer 5th (Mhow) Division.

Colonel Thomas Grainger, Indian Medical Service, Principal Medical Officer, Burma Division.

Colonel Peter Broome Giles, Administrative Medical Officer 1st London Division.

Lieutenant-Colonel Herbert Ellison Rhodes James, F.R.C.S., Royal Army Medical Corps (Retired), attached General Staff, War Office.

KNIGHT COMMANDER OF ST. MICHAEL AND ST. GEORGE.

John Rose Bradford, Esq., M.D., D.Sc. Physician to University College Hospital.

COMMANDERS OF ST. MICHAEL AND ST. GEORGE.

Alan Hay Milne, Esq., Secretary to the Incorporated Liverpool School of Tropical Medicine.

Temistocle Zammit, Esq., M.D., Government Analyst, Public Health Department, Malta.

Dugald Christie, Esq., F.R.C.P., L.R.C.S., Head of the Medical Missionaries in China.

KNIGHT BACHELOR.

The Hon. John McCall, M.D., Agent-General in London for Tasmania, and formerly President of the Central Board of Health.

KNIGHT COMMANDER VICTORIAN ORDER.

Bertrand Dawson, Esq., M.D. Physician to the King.

COMMANDERS VICTORIAN ORDER.

Fleet Surgeon Arthur Reginald Bankart, M.V.O., R.N.

Milsom Rees, Esq., F.R.C.S.Edin. Laryngologist to the King.

Mayo Robson, Esq., D.Sc., F.R.C.S.

MEMBER VICTORIAN ORDER (FOURTH CLASS).

William Fairbank, Esq., M.R.C.S. Senior Surgeon to H.M.'s Household at Windsor Castle.

